# Three-dimensional changes in regional right ventricular curvature and function in tetralogy of fallot

**DOI:** 10.1186/1532-429X-17-S1-P214

**Published:** 2015-02-03

**Authors:** Waseem Cossor, Francesco Maffessanti, Karima Addetia, Victor Mor-Avi, Keigo Kawaji, David A Roberson, Karin E Dill, Peter Varga, Roberto Lang, Amit R Patel

**Affiliations:** Pediatric Cardiology, Advocate Children’s Hospital, Chicago, IL USA; Cardiology, University of Chicago, Chicago, IL USA; Pediatric Cardiology, University of Chicago, Kragujevac, IL USA; University of Chicago, Chicago, IL USA

## Background

Many patients with Tetralogy of Fallot (TOF) have residual pulmonic stenosis and/or regurgitation after surgical repair, leading to maladaptive right ventricular (RV) remodeling. Cardiovascular magnetic resonance (CMR) is currently the gold standard for evaluating RV size and function. Analysis of regional volume and three-dimensional (3D) curvature of the RV from CMR images may provide new insights into the remodeling process. The aim of this study is to describe RV remodeling by characterizing regional endocardial surface curvature and function in patients with TOF.

## Methods

CMR (1.5T Philips Achieva) was performed in subjects with repaired TOF (N=17, age range 9-53 years) and healthy volunteers (N=10, age range 23-43 years). The RV end-diastolic and end-systolic endocardial surfaces were manually segmented from a contiguous stack of short axis cine slices to construct a 3D model using custom software (figure). This model was used to measure regional volume and ejection fraction for the RV inflow, outflow, and trabecular regions. Local endocardial surface curvature was displayed on a color map of the 3D volume surface and regional curvature was calculated for the RV inflow, outflow, trabecular, free wall, and septal segments. The values for curvature were indexed to volume to remove the influence of volume between regions. The parameters from individuals with TOF were compared to those acquired from the healthy volunteers.

**Figure 1:**
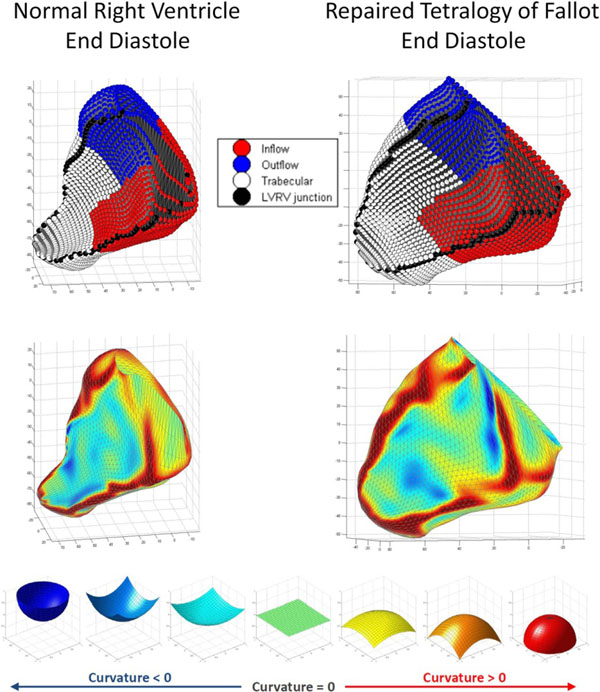
Right ventricle 3D models depicting regional volume (upper) and local surface curvature (lower). Curvature scale ranges from -1 (perfect concave half-sphere) to +1 (perfect convex half-sphere).

**Figure:**
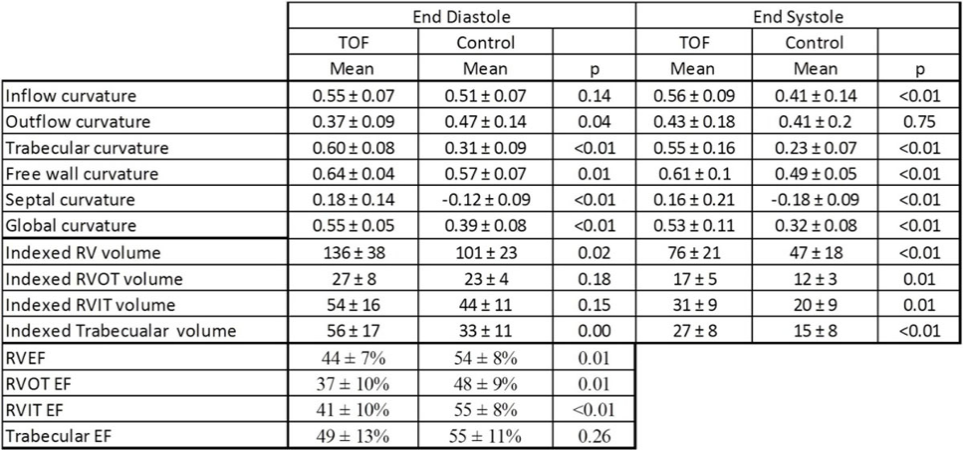
Figure 2

## Results

As shown in the table, individuals with TOF had larger global RV volume and reduced ejection fraction when compared to controls. The trabecular region had relatively preserved ejection fraction, despite exhibiting the most enlargement. Conversely, the outflow and inflow regions had greater reductions in ejection fraction but only mild enlargement. The Trabecular region enlargement coincided with increased regional curvature, which was not as striking in the inflow and outflow regions. Those with TOF had higher overall free wall curvature. Septal curvature in TOF patients was higher (slightly convex) compared to controls (slightly concave). Global curvature, combining all septal and free wall regions, was higher in TOF patients.

## Conclusions

Using analysis of RV 3D regional curvature and volumes from CMR images, we show that individuals with repaired TOF have regional variation in RV remodeling and function. Further evaluation is required to understand the clinical implications of this variable remodeling.

